# ‘Self-collected upper respiratory tract swabs for COVID-19 test’: A feasible way to increase overall testing rate and conserve resources in South Africa

**DOI:** 10.4102/phcfm.v12i1.2445

**Published:** 2020-05-28

**Authors:** Adeloye A. Adeniji

**Affiliations:** 1 Division of Family Medicine and Primary Care, Faculty of Health Sciences, Stellenbosch University, Cape Town, South Africa; 2 Ceres Hospital, Cape Winelands Health District, Department of Health, Ceres, South Africa

**Keywords:** nasal, nasopharyngeal, oropharyngeal, South Africa, World Health Organization (WHO), National Institute for Communicable Diseases (NICD), personal protective equipment (PPE), person under investigation (PUI)

## Abstract

Disparity in the testing rate of SARS-CoV-2 amongst different countries and regions is a very big challenge in understanding the COVID-19 pandemic. Although some developed countries have a very high testing rate and subsequently a high number of confirmed cases, less developed countries have a low testing rate and an illusive positivity rate. Collection of the upper respiratory specimen is not often comfortable. The discomfort could be accompanied with epistaxis and headache in some patients. The trained personnel taking the swab is forced to protect self with personal protective equipment (PPE) to avoid infections that may result from the patient due to provoked cough, sneezing and spitting. This study looks into an efficient means of increasing the testing rate for COVID 19 without compromising the quality. A literature review was conducted on the different modalities of collecting upper respiratory specimens and assessing the efficacy of samples collected using different methods in terms of the laboratory yield of different pathogens. Self-collection of upper respiratory tract specimen for diagnostic purposes is not new. Studies have demonstrated that trained staff-collected nasal swabs are not in any way superior to self-collected or parent-assisted swabs. The laboratory yield of different specimens is not determined by who took the sample but by the anatomical site from where the specimen was collected. Self collection of the upper respiratory swabs will not only increase the testing rate but also preserve the scarce PPE and reduces health care worker’s COVID 19 infection rate in South Africa.

## Introduction

The World Health Organization (WHO) on 11 March 2020 declared COVID-19 a global pandemic after more than 118 000 confirmed patients were reported in over 114 countries with 4291 fatalities.^1^ Of these, 90% of the reported cases were from four countries, 57 countries reported 10 or less cases and 81 countries did not report any case.^1^

This pronouncement was met with different regional and national reactions to curtail this epidemic within a manageable perimeter. South Africa and some of the African countries adopted national lockdown with social distancing and elaborate testing for COVID-19.

South Africa has shown a strong political will regarding the management of this epidemic within her shores. The South African government led by example in structures and implementation of regulations to enhance a speedy return to normalcy.

On 23 March 2020, President Cyril Ramaphosa announced the decision of the National Coronavirus Command Council. The decision was to enforce a 21-day nation-wide lockdown starting from 26 March 2020^2^ (this was eventually extended for another 2 weeks on 09 April 2020). This announcement was probably provoked by the progression of confirmed cases during the preceding weeks. The confirmed cases rose from 61 to 402 within a period of 8 days.^2^ It was confirmed that South Africa had conducted 12 815 tests for COVID-19, of which 10 803 were carried out in private laboratories and 2012 were effected in government laboratories.^3^

In terms of population coverage, South Africa’s health system is dominated by the public health sector, whilst the private health sector mainly caters to the affluent members of the society.^4^ The above numbers show that a big proportion of South Africa’s public health facility users were not tested for COVID-19.

Considering the resources involved, the cost of mass testing could be beyond the reach of the government. Human resources and the availability of personal protective equipment (PPE) are a big global challenge, and even daunting in the African context. There is a need to adopt burden-sharing and prioritise the use of limited resources in a manner that would help to meet Africa’s medical needs.

The family medicine principle of Evidence-Based Management of Resources to meet index circumstances in healthcare delivery is very important in this pandemic.

## Persons under investigation Coronavirus disease 2019 (COVID-19)

The National Institute of Communicable Diseases (NICD) has lived up to the expectations by providing timely information and guidelines for the management of this epidemic. This includes the case definition of persons under investigation (PUI), which are being reviewed constantly from the initial phase of the epidemic to the present one, in which a substantial amount of community lateral transfer has been confirmed. On 02 April 2020, the criteria for PUI was defined as persons with acute respiratory illness with sudden onset of at least one of the following symptoms: cough, sore throat, shortness of breath, fever equal to or more than 38 °C (measured) or history of fever (subjective) irrespective of admission status.^5^

The present case definition of PUI has placed an enormous amount of pressure on the already constrained public health system. The definition means that all acute asthmatics and acute chronic obstructive pulmonary disease (COPD) patients must be tested. All patients presenting with flu-like symptoms are supposed to be tested for COVID-19 according to the present case definition of PUI.

Compounding the existing issues around the availability of PPE for healthcare workers is the needed bed spaces for the purpose of isolation and management of moderate to severe cases if this case definition is to be implemented in the public health system.

## Testing rate for COVID-19

In South Africa, the diagnosis of COVID-19 is made by a positive laboratory test for SARS-CoV-2 by polymerase chain reaction (PCR). Works are in progress for using other molecular tests.

**FIGURE 1:**
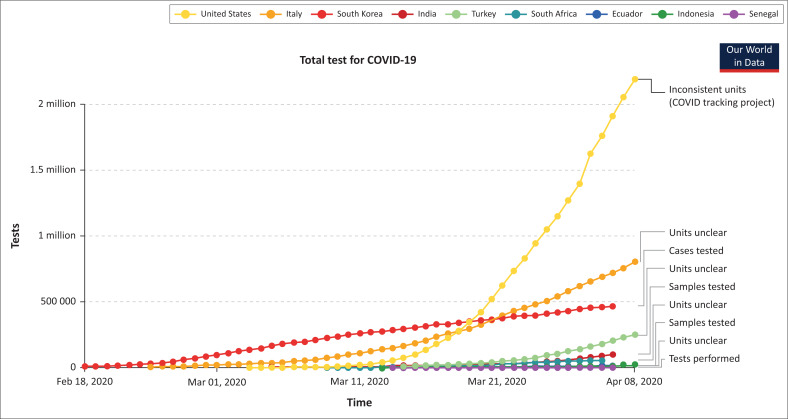
Total tests performed for COVID-19 across selected countries as of 08 April 2020.^6^

**FIGURE 2:**
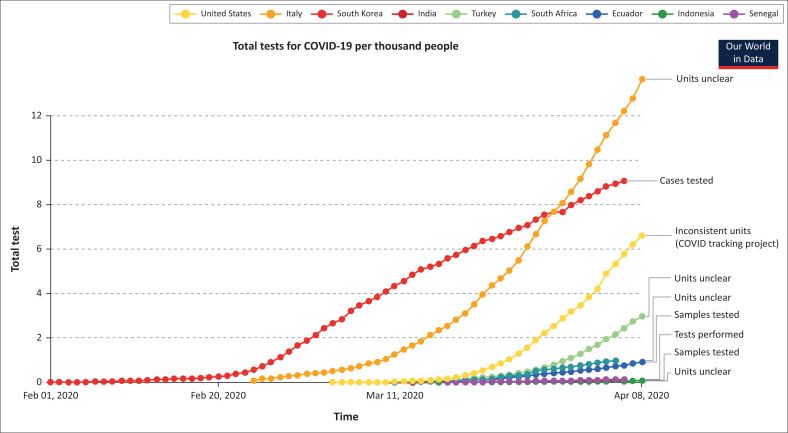
Total tests for COVID-19 per 1000 persons across selected countries as of 08 April 2020.^6^

South Africa and Senegal are the two African countries represented in charts depicted in Figures 1 and 2; however, the testing rate in these countries was still not impressive. With a testing rate of 0.97 per 1000 people and a total of 56 873 people tested as of 05 April 2020, South Africa has the highest testing rate in sub-Saharan Africa.^6^ This is not comparable with the testing rate of 13.6 per 1000 people in Italy and a total of 807 125 people tested as of 08 April 2020.^6^

## Efficacy of upper respiratory tract samples collected by different methods

Various studies have examined the detection of pathogens from different anatomical sites in the upper and lower respiratory system. Various methods have been employed to collect these samples: trained staff-collected nasal (N) swabs, nasopharyngeal (NP) swabs, oropharyngeal (OP) swabs, nasal wash (NW), self-collected N swabs and parent-assisted foam N swabs.

Stephanie A. Irvin and colleagues compared N and NP swabs for influenza detection in adults. They found that of the 240 adults, 33 (14%) tested positive for influenza by real-time polymerase reaction (rRT-PCR).^7^ Using rRT-PCR, the sensitivity of N swabs was 89% (95% Confidence Interval [CI], 78% – 99%) and that of NP swabs was 94% (95% CI, 87% – 100%).^7^ This study showed that test sensitivity between N and NP swabs did not vary significantly by swab type.

Another, the study concluded that OP sampling is significantly superior to NP sampling in detecting *S. pneumoniae* carriage in patients aged 15–19 years, and strongly supports the use of trans-oral collection when studying adolescent carriage.^8^

In 2013, an American family physician Angela P. Campbell and colleagues compared the efficacy of self-collected N swabs and NWs. Self-collected foam N swabs were obtained after instillation of saline spray and were compared with NW of 146 immunocompetent subjects of upper respiratory tract infections (URIs); sensitivities for respiratory virus detection by rRT-PCR were 95% and 88%, respectively (*p* = 0.06).^9^ Sensitivities of N swabs collected with and without saline spray in 142 URIs from immunocompetent subjects were 96% and 86% (*p* = 0.004), respectively.^9^

In 2017, German national cohort published a multi-centre study that looked into acceptability, preference and feasibility of collecting N and OP swabs, followed by microbiome analysis, in a population-based study comprising 524 participants. This study showed that microbial community structures did not differ between staff-collected and self-collected N swabs;^10^ it was also noted that self-collection of N swabs at home could be used to reduce cost and resources needed, particularly when serial measurements are to be conducted.^10^

It is worth mentioning that a self-collected sample may suffer with regard to acceptable efficacy if the patient is not well informed about the processes of collection. Literacy, age and social circumstances could play a big role in the integrity of home-based, self-collected samples.

## Recommendations to increase the COVID-19 testing rate in South Africa

A simple, comfortable, sensitive, reliable and home-based, self-collected method of swab collection is needed to increase the testing rate for COVID-19 in South Africa. Parent-assisted or self-collected swab is very much feasible in South Africa. The generic notion that swabs must be collected by trained medical personnel would not help Africa in this pandemic. There is an urgent need to review our structures and processes so that we could provide reliable data to the world about the COVID-19 epidemic in South Africa. Based on evidence, the following steps would help to increase the COVID-19 testing rate in South Africa:

1. Broad community education on self-collection of N, NP and OP swabs.
2. Distribution of swab kits to all communities in South Africa (excess to be available at South African Police Services (SAPS) and other essential facilities in the country during the lockdown period).
3. Encourage all to self-collect or opt for parent-assisted collection of the upper respiratory tract samples (NICD case definition to be strictly implemented).
4. Self-collected/parent-assisted samples to be registered and processed at the nearest clinic.
5. South Africa-based study to compare the efficacy of self-collected and trained staff-collected specimens amongst the confirmed COVID-19 patients in the country.

## Conclusion

South Africa would be able to cope with this epidemic if the doctors, nurses and other healthcare workers are relieved of the burden of swab collection. This would positively affect the number of available PPE and reduce the exposure of hospital staff to COVID-19 infection. It would not only improve compliance of social distancing rules but also enhance the availability of reliable data on COVID-19 infectivity in South Africa.
